# Spectral tuning of colloidal Si nanocrystal luminescence by post-laser irradiation in liquid†

**DOI:** 10.1039/d0ra05205a

**Published:** 2020-09-07

**Authors:** Ze Yuan, Toshihiro Nakamura

**Affiliations:** Faculty of Pure and Applied Sciences, University of Tsukuba Tsukuba Ibaraki 305-8573 Japan; Department of Electrical and Electronics Engineering, Hosei University Tokyo 184-8584 Japan nakamura@hosei.ac.jp

## Abstract

We report a simple technique to tune the luminescence spectra of blue-emitting colloidal silicon nanocrystals (Si-ncs) to the ultraviolet region *via* post-laser irradiation. The post-laser irradiation of the as-prepared colloidal Si-ncs which exhibited a broad photoluminescence (PL) centered at ∼390 nm with a bandwidth of 105 nm caused a spectral peak shift to ∼330 nm and a decrease in the bandwidth to 80 nm. The degree of the spectral tuning can be controlled by changing the irradiated laser power. The spectroscopic and transmittance electron microscopy analysis revealed that this spectral tuning effect is due to the size reduction of Si-ncs by selective absorption of post-irradiated laser light by particular-sized Si-ncs that have bandgap energies resonant with the irradiated photon energy, as well as the resultant ablation of these Si-ncs.

## Abstract 2

We report a simple technique to tune the luminescence spectra of blue-emitting colloidal silicon nanocrystals (Si-ncs) to the ultraviolet region *via* post-laser irradiation.

## Graphical abstract

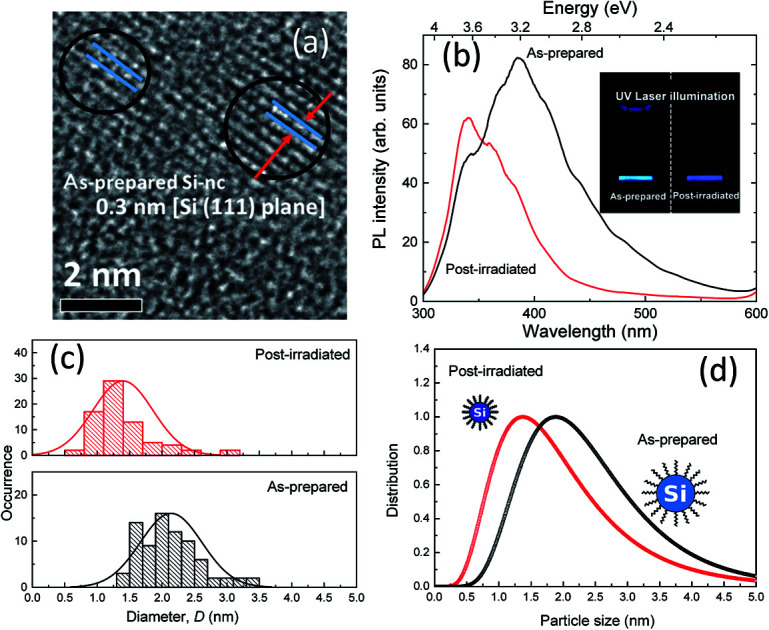

## Introduction

Semiconductors with optical response in the ultraviolet (UV) region have evoked immense interest from researchers owing to various potential applications, such as light-emitting diodes, photodetectors, and nanomedicine (bioimaging).^1–7^ Specifically, UV light emission from semiconductors can be utilized for applications in high-density information storage devices (*i.e.*, the shorter wavelength a light-emitting device operates, the more information the device can store) because the focus of UV light is more sharp than the longer wavelength light due to the diffraction limit. Moreover, because the UV light tends to produce lower heat radiation than the near-IR light, UV light-emitting devices are also suitable for medical care of heat-labile areas in the human body such as surgical procedures to correct nearsightedness.^8^ However, due to difficulties in the reliable production of high quality crystals that emit efficient UV luminescence, realizing efficient and low-cost light-emitting devices using III–V nitrides such as AlGaN and AlN remains a challenging task.

Semiconductor nanocrystals are promising materials for light emitting applications due to the controllable energy bandgap because of the quantum size effects. Among semiconductor nanocrystals, silicon nanocrystals (Si-ncs) have several unique merits, such as, nontoxicity, earth-abundance, and biocompatibility. When the physical dimensions of Si-ncs decrease below the bulk-Si exciton Bohr radius of about ∼4 nm, it exhibits visible luminescence owing to quantum confinement-induced bandgap widening. The tunability of the emission energy in Si-ncs spans from the NIR region (∼1.14 eV) to the near-UV region (∼3.26 eV).^9–11^ Recently, many research groups have proposed various fabrication processes of colloidal alkyl-terminated Si-ncs which resist oxidation without exhibiting degradation in ambient air,^12–14^ to respond to demands for the rapid development of solution-based technology.

Pulsed laser irradiation in liquid is an excellent technique to prepare luminescent colloidal nanoparticles^15–17^ such as semiconductor nanocrystals and metals.^18,19^ In the case of silicon nanocrystals, oxide-covered silicon nanocrystals are prepared by laser ablation of bulk silicon in aqueous solution^20–25^ and organic solution.^15,26–28^ In these processes, the ablation and resultant fragmentation are efficiently achieved by irradiating Si targets with high power density pulsed laser light in solvents, leading to nanoparticle formation after the generation of Si clusters in the bubbles of the solvent.^18,19^ Simultaneously, the nanoparticle surfaces are terminated with oxygen or organic ligands *via* chemical reactions with the nanoparticle surfaces. The control of size distribution of colloidal nanoparticles, including Si nanocrystals, is an important task in the laser ablation process in liquids, and various researches have revealed key factors that determine the size of nanoparticles: Intartaglia *et al.* demonstrated that laser irradiation with higher laser fluence generates larger Si nanocrystals.^20,21^ Yang *et al.* revealed a clear dependence of the size distribution on the laser fluence.^29^ Furthermore, post-treatments such as centrifugation^29^ and ultrasonification^30^ processes after the formation of Si nanocrystals enable control of the size distribution. Recently, Yang *et al.* demonstrated the formation of PbS nanocrystals with extremely low size dispersion below 10% by employing post-laser irradiation techniques.^31^ The selective absorption effect of post-irradiated laser light in larger nanocrystals causes formation of monodispersed smaller nanocrystals. This size control strategy can be applied in other semiconductor nanocrystals, including Si-nc, and it is useful for spectral tuning of the nanocrystals.

In this article, we demonstrate spectral tuning of blue-emitting colloidal alkyl-terminated Si-nc through pulsed laser ablation of porous silicon by post-laser irradiation techniques. The PL band of Si-nc centered at ∼390 nm with a bandwidth of ∼108 nm is clearly tuned in the UV region, *i.e.*, the peak position is shifted to ∼330 nm and the bandwidth is decreased to ∼80 nm. The optical and structural properties of the spectral-tuned colloidal Si-nc to the UV region are investigated in detail. These analyses reveal that the spectral tuning is due to the changes in the size distribution of Si-nc by selective absorption of larger Si-nc and the resultant ablation effects.

## Experimental

**Fig. 1:**
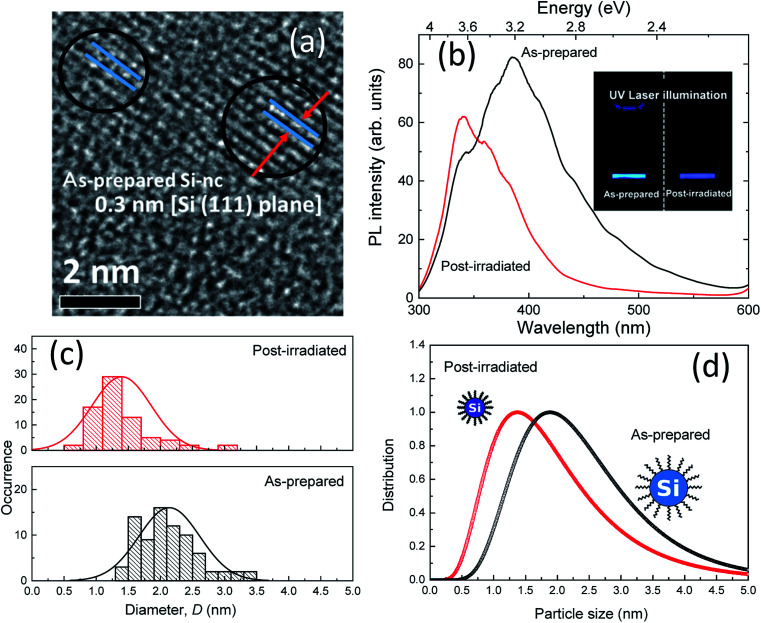
(a) High-resolution TEM image of colloidal Si-ncs prepared by the laser ablation of PSi powder in 1-octene. (b) PL spectra of as-prepared and post-laser-irradiated colloidal Si-nc samples. The inset shows photographs of the corresponding samples in 1-octene under UV laser illumination. The laser fluence of the post-laser irradiation is 0.6 J cm^−2^. (c) Size-distribution histograms of the as-prepared (top) and post-laser-irradiated (bottom) colloidal Si-nc samples estimated from the TEM image. (d) Size distributions of Si-nc evaluated from SAXS measurements.

The colloidal Si-ncs were synthesized using our previously described fabrication process,^17^ which involves laser ablation of PSi powder in an organic solvent (1-octene). First, to obtain PSi powder, metallurgical-grade polycrystalline Si powder with a mean diameter range of 3–11 μm (Vesta Ceramics) was stain-etched in a HF : H_2_O = 4 : 1 solution, to which HNO_3_ was gradually added. The solution composition was HF : H_2_O : HNO_3_ = 4 : 1 : 20 in terms of the volume ratio. The etching time was 30 min. After stain-etching, 100 mg of the prepared PSi powder was dispersed in 1-octene with a volume of 3 mL. Then, pulsed laser light at 532 nm from a Q-switched Nd:YAG laser (continuum) was irradiated with a pulse duration of 5 ns and a repetition rate of 15 Hz for 18 h. During laser irradiation, the PSi/1-octene solution was constantly stirred by a magnetic stirrer. The laser fluence was ∼1.2 J cm^−2^. After laser irradiation, the supernatant liquid was filtered by centrifugation at 13 000 rpm for 20 min with a membrane filter with a pore size of ∼200 nm. This process afforded the colloidal solution. From a representative high-resolution transmittance electron microscope (HRTEM) image, we confirmed that the obtained colloids are single crystalline Si nanoparticles with a lattice spacing of ∼0.3 nm [Si (111) plane] (Fig. 1(a)). Furthermore, we showed that the Si-ncs prepared by this process are terminated by alkyl groups from the surface chemistry analysis of our previous work.^17^ The as-prepared colloidal sample exhibited efficient blue emission under UV light illumination, as shown in the inset of Fig. 1(b). Post-laser irradiation was performed for the as-prepared colloidal Si-nc sample. In this process, we employed UV pulsed-laser light at 355 nm from a Q-switched Nd:YAG laser (a pulse duration of 5 ns and repetition rate of 15 Hz). The post-laser irradiation time was 3 h. The laser fluence range was 0.06–0.6 J cm^−2^. Note that the 1-octene was almost transparent at 355 nm, indicating that post-laser irradiation has no effect on the solution itself. As demonstrated in Fig. 1(b), the PL emission color of the post-laser-irradiated sample at 0.6 J cm^−2^ changed from blue to violet in the as-prepared sample.

Small Angle X-ray Scattering (SAXS) measurements were performed for the sample solution in a borosilicate glass capillary (diameter of 1.5 mm, WJM-Glas Müller GmbH) with an X-ray diffractometer (Smart Lab, Rigaku). The size distributions of the colloidal Si-nc samples were estimated by analyzing the SAXS data with particle size analysis software (NANO Solver, Rigaku) by a spherical scatter model. In addition, TEM (JEOL, TEM-2100) was used to obtain the size distributions. PL measurements were performed using a single monochromator equipped with a charge-coupled device (PIXIS 100B, Princeton Instruments). The fourth harmonic (266 nm) of a Nd:YAG laser (pulse duration of 5 ns, repetition rate of 15 Hz; continuum) was used as an excitation light source. Measured PL spectra were calibrated with the spectral data of a standard lamp. PLE measurements were performed using a monochromator (CT-25C, JASCO) with a Peltier-device cooled photomultiplier tube (R375, Hamamatsu) and a 50 W xenon lamp as an excitation light source. The time-resolved PL measurements were performed using a time-correlated single-photon counting module (SPC-130EM, Becker & Hickl) with a light pulse (300 ps, 2 kHz) of 355 nm from a Q-switched laser (Teem photonics) as an excitation source and a single-photon avalanche photodiode (Micro Photon Device) as a photodetector. All optical measurements were performed in a quartz cuvette at room temperature.

## Results and discussion

Fig. 1(b) shows the PL spectra of the as-prepared and post-laser-irradiated colloidal Si-nc samples. The as-prepared sample shows a broad emission band peaked at ∼390 nm (∼3.2 eV). For the post-irradiation sample, the peak energy shifts to a UV wavelength region around ∼330 nm (∼3.7 eV), and the width of the emission band decreases from ∼890 meV to ∼680 meV. These emission bands can be attributed to the quantum-confined electron–hole recombination at the band edge of Si-nc.^17^ According to this assumed recombination mechanism, its emission peak and width reflect the size-dependent bandgap energy of the Si-ncs, as reported in our previous report. However, in fact, various models have been proposed for the mechanism of blue emission in colloidal Si-ncs, such as the recombination from point defects or impurities on the surface oxides to the valence band^32–35^ and direct gap electron–hole recombination.^36,37^ The prepared Si-nc samples are considered to be terminated with alkyl species because the surface termination prepared by laser ablation depends on the type of organic solvent.^38^ We also observed the absorption peaks related to (Si–O–Si) at 1000–1200 cm^−1^,^17^ indicating that the surface of the Si-ncs is susceptible to be partially oxidized in the organic solvent through the laser irradiation process. Thus, in the present system, recombination from oxide-related defects to the valence band also occurs. In that case, the PL emission shifts from blue to the UV region; the UV emitting region (300–380 nm) is attributed to the size-dependent shift of the valence band edge.^32^ Considering any mechanisms (band-edge recombination or defect-to-valence band), we can suspect that the mechanism for the PL spectral shift is the changes in the size distribution of the colloidal Si-ncs owing to the post-laser irradiation process. It should be noted that the spectral shift may be due to the formation of additional defects by post-irradiation treatment. We will also discuss this mechanism below.

We estimated the size distributions of the colloidal Si-ncs for the as-prepared and post-laser-irradiated samples using different techniques, *i.e.*, TEM measurements and SAXS measurements. Fig. 1(c) shows the size distribution histograms obtained from the TEM images (Fig. S1†) for the as-prepared and post-laser-irradiated Si-nc samples. Although both histograms are largely dispersive, the size distribution is clearly shifted to a lower side. The size distributions estimated from the SAXS measurements, shown in Fig. 1(d), also indicate that the size distribution of post-laser-irradiated Si-nc clearly changed to a lower size region than that of the as-prepared sample. The blue-shift in the absorption spectrum shown in the ESI (Fig. S2†) also supports the size changes by post-irradiation. These experimental data support that the post-laser irradiation process causes a decrease in the size of the Si-ncs, resulting in the PL peak shift. In fact, the experimental PL peak energy shifts *versus* the colloidal Si-nc size changes are consistent with quantum confinement theory, *i.e.*, smaller colloidal Si-ncs have larger bandgap energies.^39,40^ It should be noted that the PL intensity at around 340 nm for the post-laser-irradiated sample is larger than that of the as-prepared sample [Fig. 1(b)]. This suggests that the number of smaller sized Si-ncs increases by the post-laser irradiation process, accompanying the decrease in the larger Si-ncs as understood from the size distribution results. The detailed mechanism of this size distribution change due to post-laser irradiation will be discussed below.

**Fig. 2:**
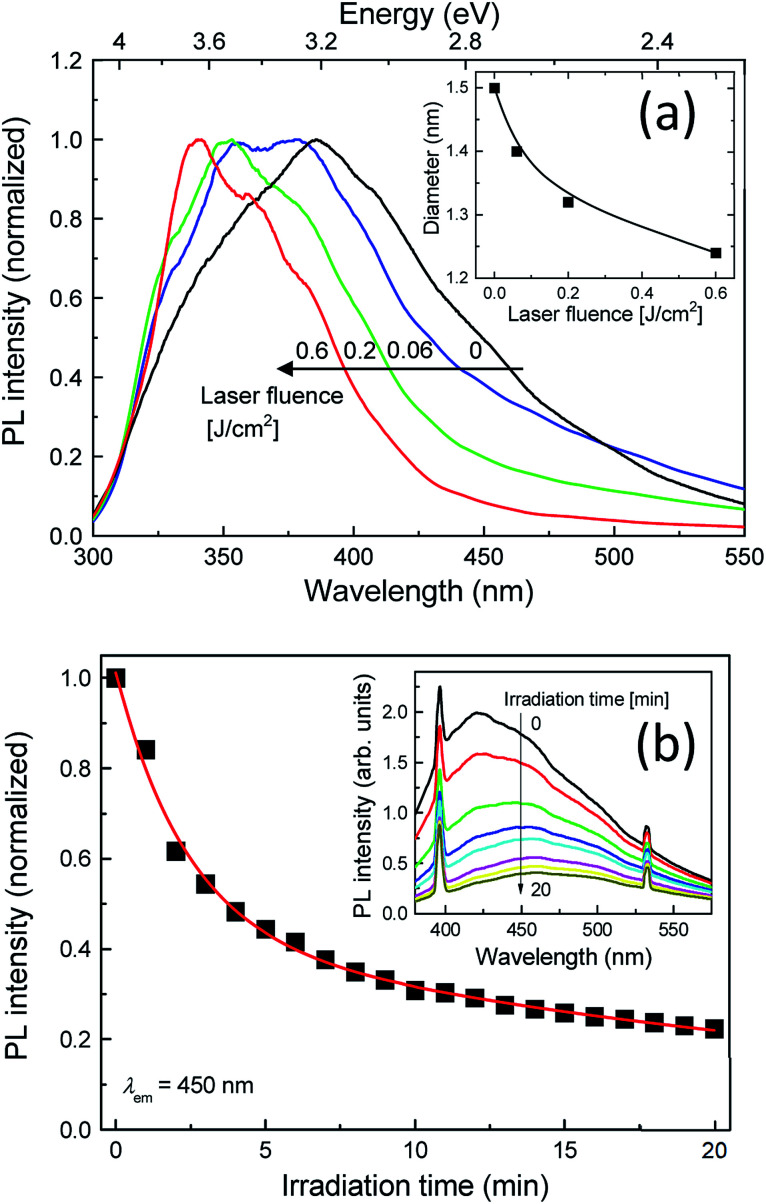
(a) PL spectra of post-laser-irradiated colloidal Si-nc samples for laser fluence, 0 (as-prepared), 0.06, 0.2, and 0.6 J cm^−2^. The inset shows the estimated diameter of Si-nc as a function of the laser fluence. (b) PL intensity of the Si-nc sample as a function of the laser irradiation time obtained from *in situ* PL measurements during the post-laser irradiation process with a pulsed laser light at 355 nm. The detected PL emission wavelength is *λ*_em_ = 450 nm. The inset shows the *in situ* PL spectra of the Si-nc sample during the post-laser irradiation. The laser fluence of the post-laser irradiation is 0.6 J cm^−2^.

In order to gain further insight into the effects of the post-laser irradiation on the spectral shapes, we prepared post-laser-irradiated colloidal Si-nc samples under different laser fluences. Fig. 2(a) shows the PL spectra of these colloidal Si-nc samples at laser fluences of 0–0.6 J cm^−2^. As the laser fluence increases, the PL peak wavelength gradually blue-shifts towards the UV region (∼330 nm), accompanying the decrease in the spectral width. This result demonstrates that emission spectral tuning of the present colloidal Si-ncs from blue to UV can be attained by changing the laser fluence. The results also indicate that the degree of the size distribution changes of Si-ncs shown in Fig. 1 depends on the laser fluence.

To quantitatively evaluate the size change degree, we obtained the sizes of the Si-ncs from the PL peak energies of the samples for different laser fluences. Assuming that the emission energy corresponds to the size-dependent bandgap energy of the Si-ncs and employing the theoretical calculation of the relation between the diameter of the Si-ncs and the bandgap energy by Wolkin *et al.*,^41^ the diameters of these samples were estimated. The inset of Fig. 2(a) shows the estimated diameters as a function of the laser fluence. As shown in the figure, the diameter decreases with the laser fluence, indicating that high power post-irradiation treatment leads to a larger size reduction.

**Scheme 1:**
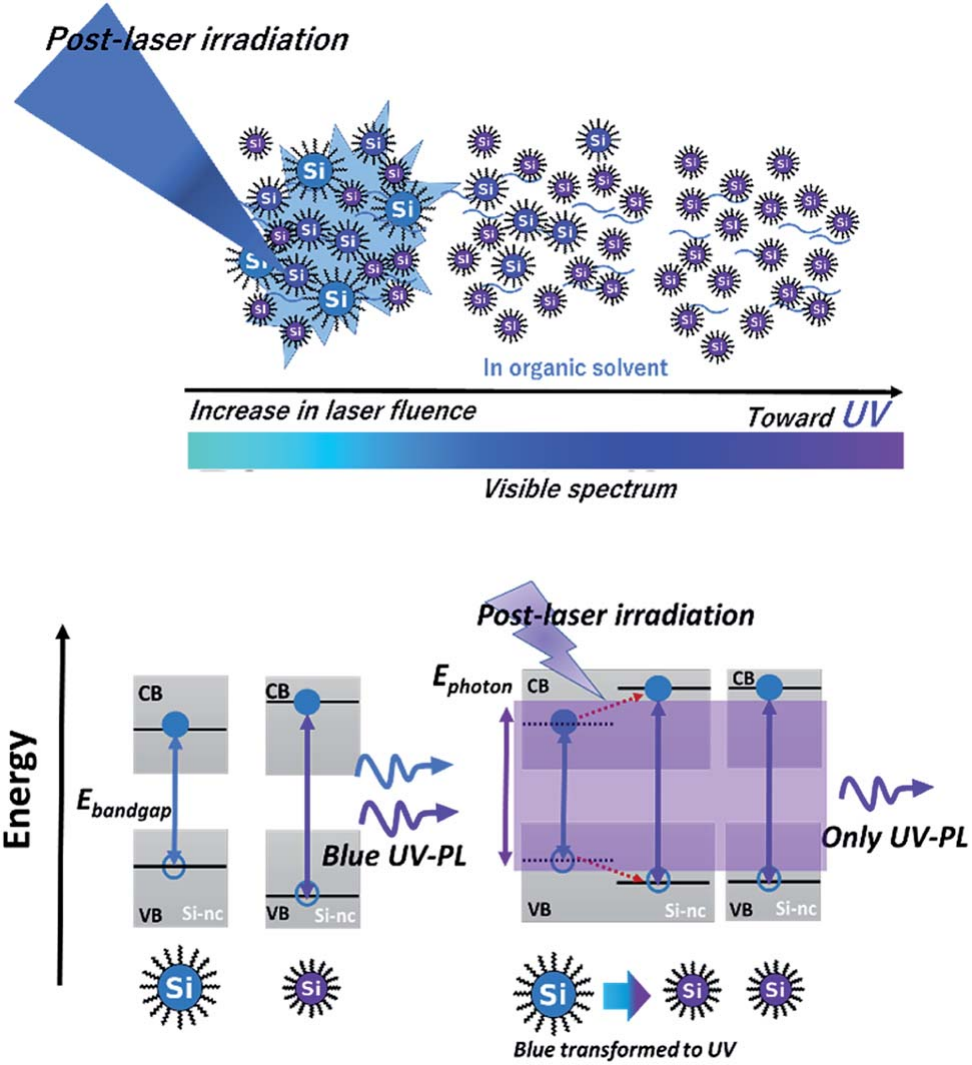
Schematic of (top) selective laser ablation of lager Si-ncs and regeneration of smaller Si-ncs from ablated species by post-laser irradiation onto the alkyl-terminated Si-nc samples dispersed in 1-octene, and (bottom) corresponding energy diagrams of these Si-ncs.

A possible origin of the post-laser-irradiation-induced size distribution changes depending on the laser fluence is the laser ablation of particular sizes of Si-ncs and the regeneration of different-sized Si-ncs from the ablated species, as illustrated in Scheme 1. The pulsed laser light at 355 nm (3.49 eV) in the post-laser irradiation process is preferentially absorbed by particular Si-ncs with bandgap energies below ∼3.49 eV, while smaller Si-ncs with larger bandgap energies are transparent to the 355 nm light. Then, laser ablation of these Si-ncs occurs due to the light-absorption-induced heat, and the ablated species are regenerated by being condensed into Si-ncs. Some of the regenerated Si-ncs, *i.e.*, larger Si-ncs with bandgap energies below ∼3.49 eV, are laser-ablated again by the post-irradiated laser light, while the size of the smaller Si-ncs remains unchanged because of their transparency to the light. After the above sequential events repeatedly occur, the size distribution finally shifts to a lower side, as shown in Fig. 1(c). This event also causes an increase in the number of smaller Si-ncs in the post-laser-irradiated sample system compared to the as-prepared one. This clearly corresponds to the experimental results in Fig. 1(b), where the PL intensity at 340 nm of the post-laser-irradiated sample is larger. Because the probability of these sequential events depends on the laser ablation efficiency, the irradiated laser fluence strongly affects the degree of the size distribution changes, as shown in Fig. 2(a).

To directly monitor the laser ablation of the larger Si-ncs and the resultant decrease in the number of the Si-ncs, we performed *in situ* PL measurements during the post-irradiation process with pulsed-laser light at 355 nm. The laser fluence was fixed at 0.6 J cm^−2^. Fig. 2(b) shows the PL intensity at 450 nm as a function of illumination time in the *in situ* PL measurements, and its inset presents the corresponding PL spectra. The sharp emission lines at 400 nm and 532 nm are the laser-induced Raman scattering peaks of the solvent (1-octene) and the second harmonic light of the Nd:YAG laser, respectively. As expected, we observed a significant decrease in the PL intensity with increasing irradiation time. This result supports the conjectured mechanism, as illustrated in Scheme 1, that the number of particular sized Si-ncs decreases due to the selective laser ablation effect.

As mentioned above, there is a possibility that the spectral shift arises from additional defect state formation. Dasog *et al.* reported that impurity-related surface states lead to the appearance of distinct emission bands depending on the impurity species.^42^ Thus, if the spectral shift is attributed to the defect states in the present case, the distinct UV band peaks should appear with increasing laser fluence. However, as shown in Fig. 2(a), the spectral change is continuous; this indicates that the defect states origin is improbable, although detailed future analysis of the defect types is needed to exactly exclude this mechanism.

**Fig. 3:**
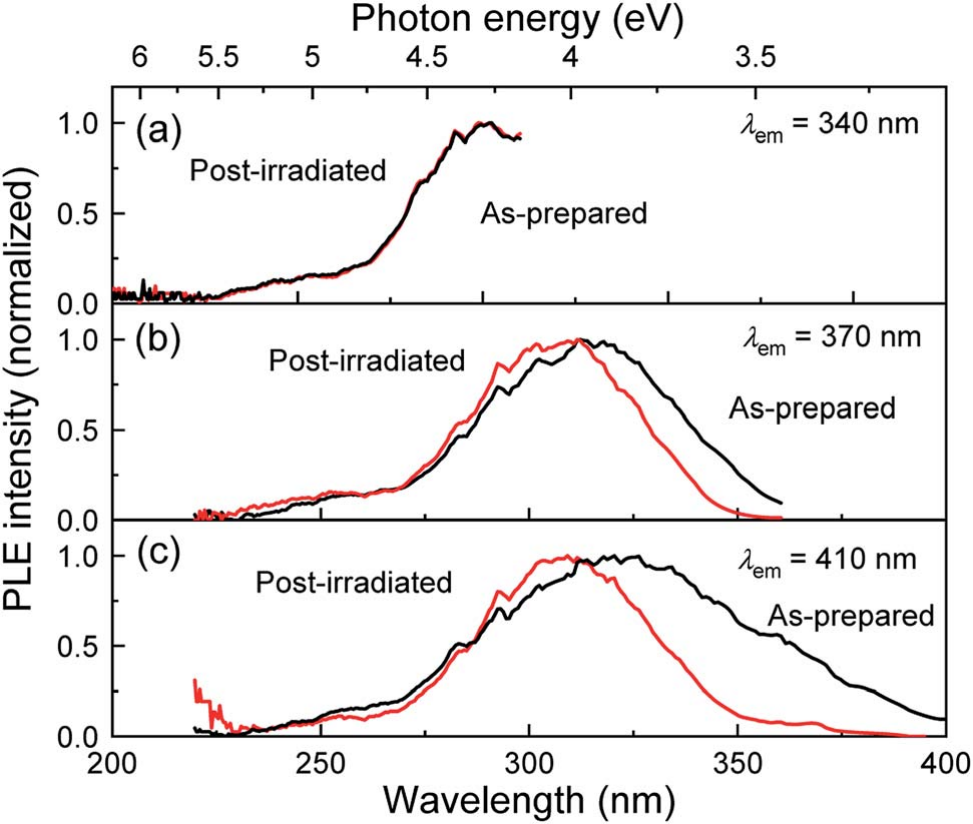
PLE spectra of the as-prepared (black curve) and post-laser-irradiated (red curve) Si-nc samples monitored at *λ*_em_ = (a) 340, (b) 370, and (c) 410 nm. The laser fluence of the post-laser irradiation is 0.6 J cm^−2^.

Fig. 3 shows the PLE spectra of the as-prepared and post-irradiated colloidal Si-nc samples dispersed in 1-octene monitored at emission wavelengths of *λ*_em_ = 340, 370, and 410 nm, respectively. The laser fluence in the post-laser-irradiated process was 0.6 J cm^−2^. As the monitored emission wavelength increases, the PLE band shifts to a longer wavelength region from 270 to 325 nm for both samples. This result reflects the fact that larger Si-ncs which emit longer-wavelength light have an absorption band at a longer wavelength side due to the size dependence of the electronic band structures in a quantum confined system (Si-ncs).^38^ By comparing the post-irradiated sample with the as-prepared sample, we can observe no spectral change at *λ*_em_ = 340 nm. However, as the emission wavelength increases, the PLE intensity of the post-irradiated colloidal Si-nc samples is significantly reduced in a blue wavelength side (325–400 nm), while in the UV wavelength region (<∼325 nm), the spectra are almost the same as that of the as-prepared sample. These results suggest that laser ablation-induced size distribution changes occur, *i.e.*, the post-laser irradiation at 355 nm light causes the decrease in the number of larger Si-ncs exhibiting the PL emission at above ∼355 nm (and having an absorption band on a longer wavelength side) due to the laser ablation of particular Si-ncs. This interpretation is consistent with the mechanism deduced from the PL spectral and size distribution measurements as discussed above.

**Fig. 4:**
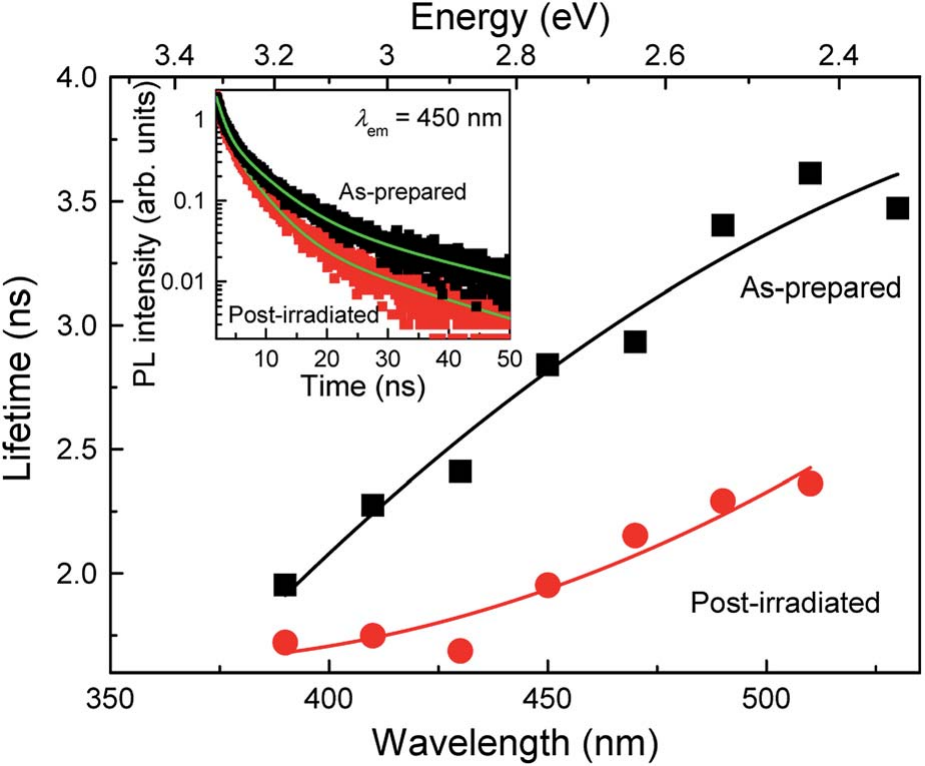
PL lifetimes of the as-prepared and post-laser-irradiated samples as a function of the emission wavelength. The inset shows the corresponding PL decay curves measured at 450 nm. The laser fluence of the post-laser irradiation is 0.6 J cm^−2^.

To evaluate the effects of post-laser irradiation on the optical transition process, we measured the PL decay curves of the colloidal Si-nc samples, as shown in the inset of Fig. 4. The decay of the post-irradiated sample is faster than that of the as-prepared sample, indicating that the non-radiative transition process increases due to the post-laser irradiation process. Fig. 4 shows the PL lifetime as a function of emission wavelength estimated from the fitting of the measured PL decay curves with the three-exponential model.^17^ The PL lifetime of the post-irradiated colloidal Si-ncs is clearly shorter than that of the as-prepared Si-ncs in the whole measured wavelength region. Thus, because the integrated PL intensity of the post-laser irradiation sample is lower, as understood from Fig. 1(b), these results indicate an increase in the non-radiative decay rate after the post-laser irradiation. To evaluate the PL efficiency changes before and after post-irradiation treatment, we estimated the relative PL quantum efficiency of the post-irradiated Si-nc sample. By employing the PL quantum efficiency data for the as-prepared sample from previous work (∼14%^17,38^), we can roughly calculate the relative quantum efficiency from the PL spectra in Fig. 1(b) and the absorbance spectra in Fig. S2 of the ESI.† By using the absorbance values (0.22 and 0.24 for as-prepared and post-irradiated Si-ncs, respectively) at 266 nm and the integrated PL intensities (9400 and 4800 in arbitrary units for the as-prepared and post-irradiated Si-ncs, respectively), we found the relative PL quantum efficiency to be ∼7% for the post-laser-irradiated Si-ncs. This result supports the increase in the non-radiative rates by post-irradiation treatment. The mechanism for the increased non-radiative rate can probably be attributed to the increase in the surface defects during the post-laser irradiation process. The defects of Si-ncs that affect the PL efficiency are known to be dangling bonds at the surface of the Si-ncs.^43–45^ There are two different types of defects, *i.e.*, the breaking bridges of the Si–Si bonds, known as *D* centers,^43,44^ and the so-called *P*_b_ centers, which are dangling bonds of Si atoms backbonded by three other Si atoms and exist at the Si/SiO_2_ interface.^45^ These defects are known to be easily formed by light-illumination. Thus, the increase in the non-radiative rate may be due to the formation of these defects, although exact evaluation of the defect types using electron spin resonance techniques is needed. The sequential events of laser ablation and regeneration of Si-ncs by post-laser irradiation as depicted in Scheme 1 may lead to insufficient surface passivation of the Si-ncs by organic species due to the existence of oxygen dissolved in the solution (1-octene). Further improvements, such as purging dissolved oxygen and/or laser irradiation under inert gas atmosphere, may suppress the increase in the non-radiative process.

## Conclusions

In conclusion, we demonstrated spectral tuning of blue-emitting colloidal alkyl-terminated Si-ncs *via* pulsed laser ablation of PSi by post-laser irradiation techniques. The blue PL band of the Si-ncs is clearly tuned in the UV region. By increasing the irradiated laser fluence, we can control the spectral tuning as the peak shifts to ∼340 nm from ∼390 nm and the bandwidth decreases to ∼80 nm from ∼105 nm. From the detailed analysis of the size distribution of the samples, we clarified that the spectral tuning to the UV region is due to the overall size reduction of the Si-ncs by post-laser irradiation. The mechanism of this size reduction is repetitive laser ablation of larger Si-ncs with bandgap energies below the post-irradiated laser light energy. To appropriately change the laser irradiation light wavelength, further precise control of the size distribution can be attained. The present prepared UV-emitting colloidal silicon nanocrystals can potentially be utilized in water disinfection applications because of the inheritance of biologically harmless silicon material. Furthermore, the post-laser irradiation process in liquid demonstrated in the present work is a potential novel route to realize the bandgap engineering of various semiconductor nanocrystals and control their emission colors *via* size distribution modification.

## Conflicts of interest

There are no conflicts to declare.

## Supplementary Material

RA-010-D0RA05205A-s001
