# Supplementary material for: Spectral tuning of colloidal Si nanocrystal luminescence by post-laser irradiation in liquid
Source: RSC Adv. 2020 Sep 7;10(54):32992–8. doi: 10.1039/d0ra05205a (PMC9056600; doi:10.1039/d0ra05205a)
Supplement: RA-010-D0RA05205A-s001 [file RA-010-D0RA05205A-s001.pdf]

Electronic Supplementary Material (ESI) for RSC Advances.
This journal is © The Royal Society of Chemistry 2020

Supplementary Information

Spectral Tuning of Colloidal Si Nanocrystal Luminescence by Post-Laser irradiation in Liquid

Ze Yuan, *Toshihiro Nakamura*

† Faculty of Pure and Applied sciences, University of Tsukuba, Tsukuba, Ibaraki 305-8573, Japan

‡ Department of Electrical and Electronics Engineering, Hosei University, Tokyo 184-8584, Japan

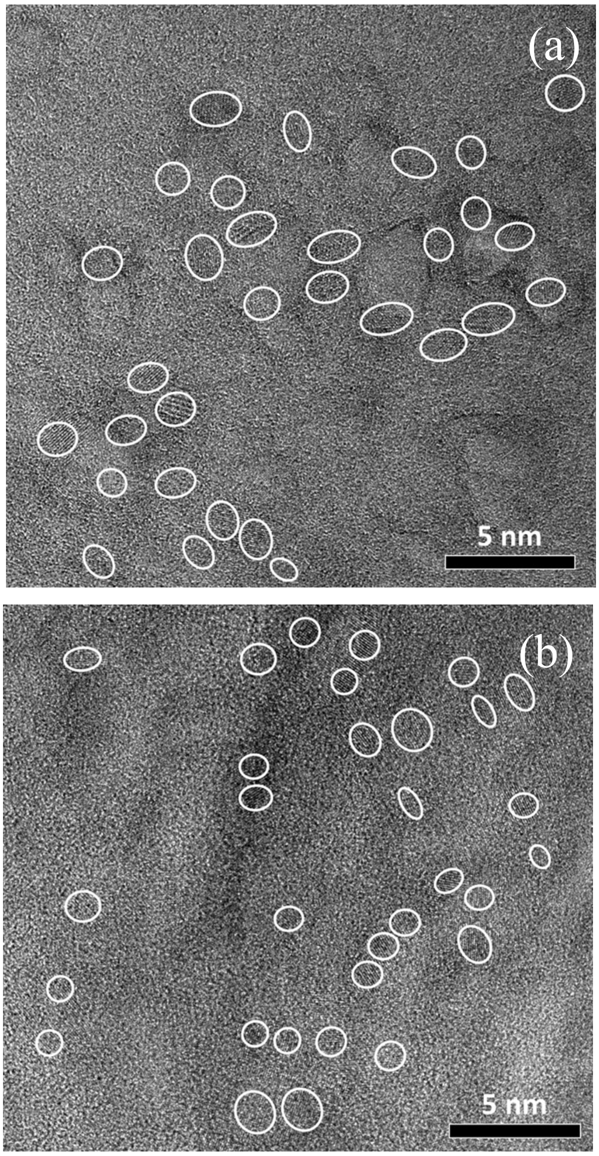

Supplementary Figure S1 | TEM images of (a) as-prepared and (b) post-irradiated colloidal Si nanocrystals.

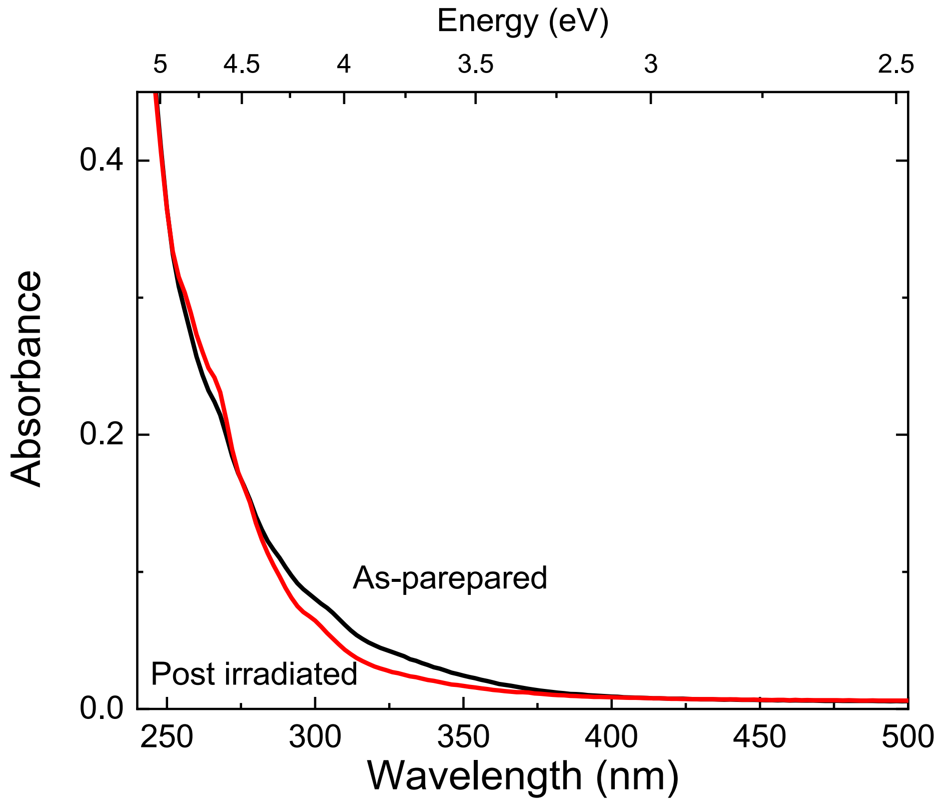

Supplementary Figure S2 | Absorbance spectra of as-prepared and post-irradiated colloidal Si nanocrystals
